# Effectiveness of complete primary vaccination against COVID-19 at primary care and community level during predominant Delta circulation in Europe: multicentre analysis, I-MOVE-COVID-19 and ECDC networks, July to August 2021

**DOI:** 10.2807/1560-7917.ES.2022.27.21.2101104

**Published:** 2022-05-26

**Authors:** Esther Kissling, Mariëtte Hooiveld, Iván Martínez-Baz, Clara Mazagatos, Naoma William, Ana-Maria Vilcu, Marjolein N Kooijman, Maja Ilić, Lisa Domegan, Ausenda Machado, Simon de Lusignan, Mihaela Lazar, Adam Meijer, Mia Brytting, Itziar Casado, Amparo Larrauri, Josephine-L K Murray, Sylvie Behillil, Brechje de Gier, Ivan Mlinarić, Joan O’Donnell, Ana Paula Rodrigues, Ruby Tsang, Olivia Timnea, Marit de Lange, Maximilian Riess, Jesús Castilla, Francisco Pozo, Mark Hamilton, Alessandra Falchi, Mirjam J Knol, Sanja Kurečić Filipović, Linda Dunford, Raquel Guiomar, Jade Cogdale, Carmen Cherciu, Tessa Jansen, Theresa Enkirch, Luca Basile, Jeff Connell, Verónica Gomez, Virginia Sandonis Martín, Sabrina Bacci, Angela MC Rose, Lucia Pastore Celentano, Marta Valenciano, I-MOVE-COVID-19 and ECDC primary care study teams, I-MOVE-COVID-19 and ECDC primary care study team (in addition to authors above), Katica Čusek Adamić, Ivana Ferenčak, Bernard Kaić, Mirjana Lana Kosanović Ličina, Danijela Lakošeljac, Ivana Mihin Huskić, Diana Nonković, Nick Andrews, Jamie Lopez Bernal, Joanna Ellis, Heather Whitaker, Thierry Blanchon,, Caroline Guerrisi,, Titouan Launay,, Shirley Masse,, Sylvie van der Werf, Vincent Enouf, John Cuddihy, Lois O’Connor, Adele McKenna, Michael Joyce, Cillian de Gascun, Joanne Moran, Rianne van Gageldonk-Lafeber, Susan J Hahné, Hester E de Melker, Ewout B Fanoy, Stijn Raven, Marit Middeldorp, Irina Kislaya, Baltazar Nunes, Rita Roquete, Adriana Silva, Aryse Melo, Inês Costa, Nuno Verdasca, Patrícia Conde, Amélia Soeiro, Maria Elena Mihai, Iulia Bistriceanu, Alina Ivanciuc, Diana Dintoi, Catalina Pascu, Adrian Jidovu, Debbie Sigerson, Diogo FP Marques, Anna Molesworth, Leanne Quinn, Miranda Leyton, Selin Campbell, Janine Thoulass, Jim McMenamin, Inmaculada Casas Flecha, Ana Martínez Mateo, Daniel Castrillejo, Eva María Martínez Ochoa, Carmen Quiñones Rubio, Concepción Delgado-Sanz, Jesús Oliva, Ana Miqueleiz, Ana Navascués, Camino Trobajo-Sanmartín, Carmen Ezpeleta, Paula López Moreno, Javier Gorricho, Eva Ardanaz, Fernando Baigorria, Aurelio Barricarte, Cristina Burgui, Enrique de la Cruz, Nerea Egüés, Manuel García Cenoz, Marcela Guevara, Conchi Moreno-Iribas, Carmen Sayón, Pasi Penttinen, Christiana Carstairs

**Affiliations:** 1 Epiconcept, Paris, France; 2 Nivel, Utrecht, the Netherlands; 3 Instituto de Salud Pública de Navarra - IdiSNA, Pamplona, Spain; 4 Consortium for Biomedical Research in Epidemiology and Public Health (CIBERESP), Madrid, Spain; 5 National Centre for Epidemiology, Institute of Health Carlos III, Madrid, Spain; 6 Public Health Scotland, Glasgow, United Kingdom; 7 INSERM, Sorbonne Université, Institut Pierre Louis d'épidémiologie et de Santé Publique (IPLESP UMRS 1136), Paris, France; 8 National Institute for Public Health and the Environment (RIVM), Bilthoven, the Netherlands; 9 Croatian Institute of Public Health, Zagreb, Croatia; 10 Health Service Executive-Health Protection Surveillance Centre, Dublin, Ireland; 11 Instituto Nacional de Saúde Dr. Ricardo Jorge, Lisbon, Portugal; 12 Nuffield Department of Primary Care Health Sciences, University of Oxford, Oxford, United Kingdom; 13 Royal College of General Practitioners Research and Surveillance Centre, London, United Kingdom; 14 *“Cantacuzino”* National Military Medical Institute for Research and Development, Bucharest, Romania; 15 The Public Health Agency of Sweden, Stockholm, Sweden; 16 Unité de Génétique Moléculaire des Virus à ARN, UMR 3569 CNRS, Université Paris Diderot SPC, Institut Pasteur, Paris, France; 17 CNR des virus des infections respiratoires, Institut Pasteur, Paris, France; 18 National Centre for Microbiology, Institute of Health Carlos III, Madrid, Spain; 19 Laboratoire de Virologie, Université de Corse-Inserm, Corte, France; 20 National Virus Reference Laboratory, University College Dublin, Dublin, Ireland; 21 UK Health Security Agency, United Kingdom; 22 Subdirección General de Vigilancia y Respuesta a Emergencias de Salud Pública, Agencia de Salud Pública, Catalunya, Spain; 23 European Centre for Disease Prevention and Control, Stockholm, Sweden; 24 The members of the group are listed under Collaborators

**Keywords:** COVID-19, SARS-CoV-2, vaccine effectiveness, multicentre study, test-negative design, Europe, Delta variant

## Abstract

**Introduction:**

In July and August 2021, the SARS-CoV-2 Delta variant dominated in Europe.

**Aim:**

Using a multicentre test-negative study, we measured COVID-19 vaccine effectiveness (VE) against symptomatic infection.

**Methods:**

Individuals with COVID-19 or acute respiratory symptoms at primary care/community level in 10 European countries were tested for SARS-CoV-2. We measured complete primary course overall VE by vaccine brand and by time since vaccination.

**Results:**

Overall VE was 74% (95% CI: 69–79), 76% (95% CI: 71–80), 63% (95% CI: 48–75) and 63% (95% CI: 16–83) among those aged 30–44, 45–59, 60–74 and ≥ 75 years, respectively. VE among those aged 30–59 years was 78% (95% CI: 75–81), 66% (95% CI: 58–73), 91% (95% CI: 87–94) and 52% (95% CI: 40–61), for Comirnaty, Vaxzevria, Spikevax and COVID-19 Vaccine Janssen, respectively. VE among people 60 years and older was 67% (95% CI: 52–77), 65% (95% CI: 48–76) and 83% (95% CI: 64–92) for Comirnaty, Vaxzevria and Spikevax, respectively. Comirnaty VE among those aged 30–59 years was 87% (95% CI: 83–89) at 14–29 days and 65% (95% CI: 56–71%) at ≥ 90 days between vaccination and onset of symptoms.

**Conclusions:**

VE against symptomatic infection with the SARS-CoV-2 Delta variant varied among brands, ranging from 52% to 91%. While some waning of the vaccine effect may be present (sample size limited this analysis to only Comirnaty), protection was 65% at 90 days or more between vaccination and onset.

## Introduction

Coronavirus disease (COVID-19) has caused considerable morbidity and mortality with more than 66 million cases and 1.2 million deaths reported in the World Health Organization European Region as at 5 September 2021 [1]. The highly transmissible Delta variant (Phylogenetic Assignment of Named Global Outbreak (Pango) lineage designation B.1.617.2 and the AY sublineages) of severe acute respiratory syndrome coronavirus 2 (SARS-CoV-2) dominated over the Alpha variant (B.1.1.7) in Europe from July 2021 onwards and accounted for more than 99% of sequenced samples in weeks 35–36 in 2021 [2-4]. From mid-March 2021, four COVID-19 vaccines were authorised in the European Union by the European Medicines Agency: two mRNA vaccines (Comirnaty and Spikevax) and two adenoviral vector vaccines (Vaxzevria and COVID-19 Vaccine Janssen) [5]. Before the circulation of the Delta variant, randomised controlled trials indicated a high efficacy for these vaccines [6-9]. Observational post-authorisation studies in Europe are therefore important to measure the effectiveness of the different COVID-19 vaccines against the currently circulating Delta variant.

Recent discussions around recommendations for booster doses, for various populations, highlight the need to measure vaccine effectiveness (VE) by time since vaccination. Many studies have reported a decrease in VE against infection with increasing time since vaccination [10-14], and a recent review indicated that VE against for symptomatic COVID-19 disease decreased by 24.9 percentage points among all ages from 1 month to 6 months after full vaccination [15]. It can be difficult to disentangle potential changes in VE by time since vaccination from changes in VE that are due to circulation of different variants over time. The I-MOVE-COVID-19 and European Centre for Disease Prevention and Control (ECDC) networks in Europe carry out COVID-19 VE studies, including a VE study at primary care/community level [16]. All studies are based on a common generic protocol [17].

We conducted a study in symptomatic individuals swabbed for a COVID-19 test at primary and care/community level in July and August 2021, assuming that any identified COVID-19 cases were infected with the Delta variant given its dominance in Europe during this period. We estimated VE against SARS-CoV-2 symptomatic infection, by vaccine brand, age group and time since vaccination to understand if there may be waning of vaccine-induced protection over time.

## Methods

### Study design

**Figure 1:**
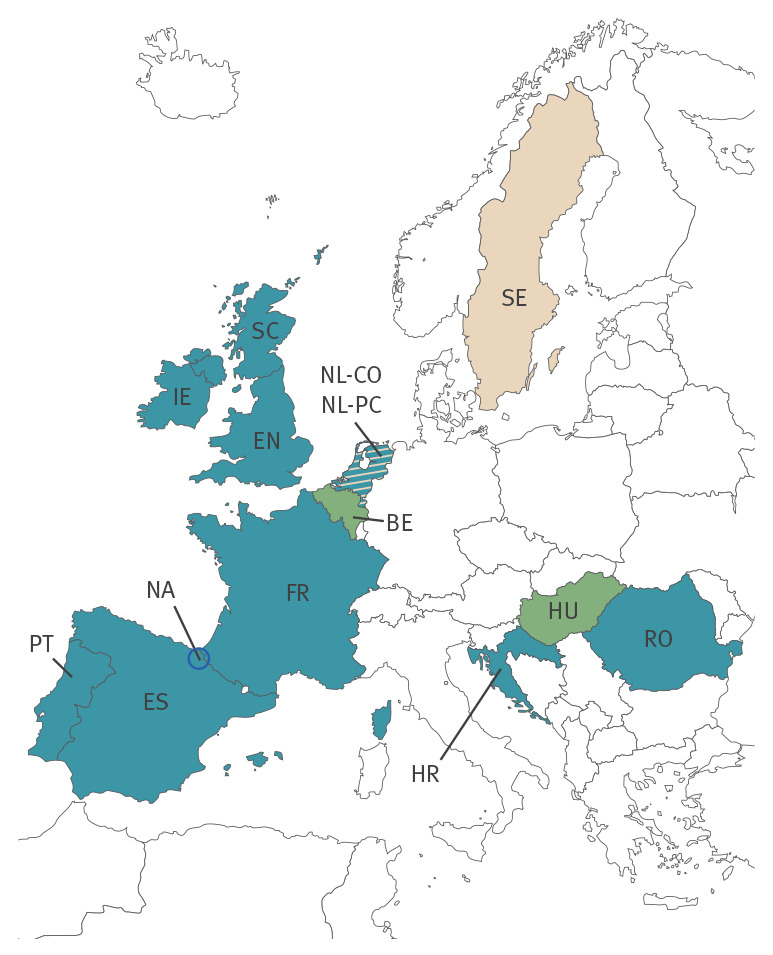
Countries and study sites taking part in the I-MOVE-COVID-19 and ECDC studies on COVID-19 vaccine effectiveness at primary care/community level, Europe, July–August 2021 (n = 14 sites)

BE: Belgium; COVID-19: coronavirus disease; ECDC: European Centre for Disease Prevention and Control; EN: England; ES: Spain; FR: France; HR: Croatia; HU: Hungary; IE: Ireland; NA: Navarre; NL-CO: the Netherlands community-based study; NL-PC: the Netherlands primary care; PT: Portugal; RO: Romania; SC: Scotland; SE: Sweden.

Ten of 14 primary care/community study sites participated in this analysis: Croatia (HR), France (FR), Ireland (IE), the Netherlands (community testing: NL-CO), Portugal (PT), Romania (RO), three regions in Spain (ES), the Navarre region in Spain (NA), as well as England (EN) and Scotland (SC) in the United Kingdom (UK) (Figure 1).

Blue: Study sites part of the vaccine effectiveness analysis (EN, ES, FR, HR, IE, NA, NL-CO, PT, RO, SC); green: study sites that did not provide data before the end of the study period (BE, HU); beige: study sites that provided data but could not be included in the analysis (no cases in study period (NL-PC) or discontinuation of surveillance during study period (SE).

We used the test-negative design to estimate VE against symptomatic infection [18]. Cases were individuals testing positive and controls were individuals testing negative for SARS-CoV-2. Study sites adapted a generic study protocol to their country-specific setting [17]. In eight of the 10 participating study sites, a sentinel physician recruited patients. The site SC used a combination of physician-based and community-based swabbing, and NL-CO used a purely community-based swabbing approach. Sites included all or a systematic sample of individuals with acute respiratory infection (ARI), defined as sudden onset of symptoms of at least one of the following: cough, sore throat, shortness of breath or runny nose; in FR, the definition was sudden onset of fever (or feeling of fever) and respiratory signs. They also included individuals who contacted the sentinel physician/swabbing centre and presented with COVID-19 symptoms, defined by at least one of the following symptoms: fever, cough, shortness of breath and sudden onset of anosmia/ageusia; in some countries, runny nose was also included in the case definition. The site NA included all individuals presenting to the Navarre Health Service primary care physicians. The site SC included a systematic sample of individuals presenting to community-attended COVID-19 centres, where both self-swabbing and swabbing by clinical staff were performed. In NL-CO, participating individuals were self-selected (see Supplementary Table S1 for characteristics of the study sites).

Demographical, clinical (age, sex and chronic conditions) and information on COVID-19 vaccination (number of doses, brand and date for each dose) was collected, via questionnaire, electronic medical records and vaccine registry linkage (the individual sources per site are listed in Supplementary Table S1).

Five study sites (EN, HR, IE, RO, SC) tested individuals exclusively by RT-PCR, five study sites (NL-CO, ES, FR, NA, PT) included individuals tested by RT-PCR or rapid antigen test. Four study sites (HR, IE, NA, RO) had sequencing results of the whole or partial genome of viruses confirmed by RT-PCR. The sites HR, IE, RO attempted to sequence all viruses, and NA sequenced a proportion of viruses, selected independently of vaccination status. Phylogenetic analysis was performed to identify the Pango lineage based on the classification v.3.1.16 2021.

### Study period

The study period included swab dates in July and August 2021, as this is when the Delta variant was dominant in participating study sites [19,20].

### Study inclusion criteria

We included individuals aged 30 years and older who belonged to their country’s age-specific target group for vaccination at time of swabbing. Where known, we excluded individuals with contraindications for vaccination, those living in a residential care facility, controls who had previously tested positive for SARS-CoV-2 and controls who had tested positive for seasonal coronaviruses. We further excluded those who were swabbed more than 10 days (RT-PCR tests) or 5 days (rapid antigen tests) after symptom onset. We excluded those with a time interval between doses not recommended by the vaccine manufacturer (< 21 days for Comirnaty, < 28 days for Spikevax/Vaxzevria, < 21 days if vaccine brand was unknown) and those with onset within 1–13 days of the first (if COVID-19 Vaccine Janssen) or second dose (for two-dose vaccines) of COVID-19 vaccine.

### Definitions of vaccination

We defined persons as completely vaccinated 14 days after receiving either the second of two recommended doses of a two-dose vaccine or a single dose of COVID-19 Vaccine Janssen. Persons were considered unvaccinated if they did not receive any COVID-19 vaccine or were vaccinated on or after the day of onset of symptoms. Persons partially vaccinated or those with an additional dose were excluded from the analysis.

For the brand-specific analysis, we restricted the data to the period when the brand was available in the country by age group. For two-dose vaccines, we only included people vaccinated with the same brand for both doses.

### Statistical analysis

We compared the odds of vaccination between cases and controls. We used logistic regression and calculated VE as 1 minus the OR expressed as a percentage. We included study site as a fixed effect and date of swab (modelled as a restricted cubic spline or as a categorical variable of swab week) in both crude and adjusted VE analyses, as time is an integral part of the test-negative design and study site is an integral part of a multicentre study.

We further adjusted for age group, sex and presence of at least one of the six commonly collected COVID-19-relevant chronic conditions (diabetes, chronic lung disease, immunodeficiencies, heart disease, renal disease and liver disease). In SC, the question “Did you receive a letter asking you to shield?” was used as a proxy for “presence of chronic condition”, assuming those not answering the question were not shielding.

In NA where symptom onset date was not available and in two study sites with ≥ 20% of missing information (IE and PT), we imputed it as 3 days before the swab date, 3 days being the median between onset and swab date at the sites where information was available.

For the age-specific analyses for all vaccines, we stratified the data into the following age groups: 30–44, 45–59, 60–74 and ≥ 75 years. For the analyses among those with a chronic condition, vaccine brand and by time since vaccination, we stratified the data into the age groups 30–59 and ≥ 60 years.

We modelled VE by time since vaccination using an interaction between vaccination and time since vaccination as a restricted cubic spline with four knots at 0 and 20 days and at the 45th and 90th centile, based on a priori knots and adaptations of Harrell’s percentiles [21]. We also measured VE by days between vaccination and onset of symptoms with a stratified analysis using monthly cut-offs: < 30, 30–59, 60–89, 90–119 and ≥ 120 days, or ≥ 90 days where sample size was small. We superimposed these values on the graph of the model to provide a validation of the modelling. We did not compute VE if one of these strata had less than 100 individuals.

We conducted sensitivity analyses excluding the study site without systematic selection of individuals for swabbing (NL-CO), excluding the largest study site (NA), varying the imputed onset dates between 2 and 5 days, including only those swabbed within 7 days of symptom onset (RT-PCR tests), including only those tested by RT-PCR, and varying the number and position of knots in the time since vaccination analysis.

## Results

### Descriptive analysis

**Figure 2:**
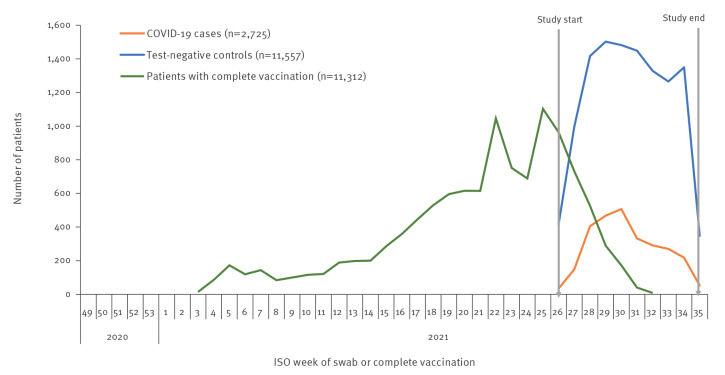
Number of cases and controls by week of swab, and by week of complete COVID-19 vaccination, I-MOVE-COVID-19 and ECDC primary care and community multicentre networks, Europe, July–August 2021 (n = 14,282)

COVID-19: coronavirus disease; ECDC: European Centre for Disease Prevention and Control; ISO: International Organization for Standardization.

After applying the exclusion criteria, we included 14,282 individuals, of whom 2,725 were cases and 11,557 were controls (Supplementary Figure S1 details the flow of exclusions). Among the cases and controls, 11,312 were completely vaccinated. Vaccination roll-out continued as cases and controls were selected (Figure 2).

Cases and controls received their second dose (for two-dose schedules) or first dose (for COVID-19 Vaccine Janssen) of COVID-19 vaccination up to 213 days before symptom onset (see Supplementary Figure S2 for time between complete vaccination and symptom onset for all individuals). Among cases, 84% (1,352/1,606) and among controls, 89% (8,632/9,706) presented with symptoms < 120 days after complete vaccination. The median days between last vaccination and symptom onset among those younger than 60 years was 50 days (interquartile range (IQR): 36–72) for cases and 42 days (IQR: 27–62) for controls. The median days between last vaccination and symptom onset among people 60 years and older was 82 days (IQR: 57–107) for cases and 78 days (IQR: 52–103) for controls.

Among controls, 35% were 60 and older compared with 24% of cases, 59% were female (vs 54% of cases), and 31% (vs 23% of cases) presented with at least one chronic condition (diabetes, heart disease, chronic lung disease, immunodeficiency, renal disease or liver disease) (Table 1).

**Table 1:** Descriptive analysis of COVID-19 cases and controls in the I-MOVE-COVID-19 and ECDC primary care and community multicentre network pooled analysis, Europe, July–August 2021 (n = 14,282)

Characteristic	SARS-CoV-2 cases (n = 2,725)		Test-negative controls (n = 11,557)	
	n	%	n	%
Age group (years)				
30–44	1,066	39	3,273	28
45–59	992	36	4,233	37
60–74	410	15	2,412	21
≥ 75	257	9	1,639	14
Sex				
Female	1,483	54	6,807	59
Male	1,242	46	4,750	41
Presence of chronic condition^a^				
Presence of chronic condition	628	23	3,626	31
No chronic condition	2,097	77	7,931	69
COVID-19 vaccination status at time of onset of symptoms				
Unvaccinated	1,119	41	1,851	16
Completely vaccinated	1,606	59	9,706	84
COVID-19 vaccination products among vaccinated				
2 doses Comirnaty (mRNA)	970	60	6,430	66
2 doses Spikevax (mRNA)	58	4	839	9
2 doses Vaxzevria (viral vector)	301	19	1,554	16
1 dose Janssen (viral vector)	259	16	724	7
2 doses Curevac (mRNA)	0	0	1	0
2 doses Coronavac (invactivated)	0	0	1	0
1 or both doses of unknown brand	9	1	80	1
2 doses Janssen (viral vector)	2	0	3	0
2 heterologous doses	7	0	74	1
Type of test				
RT-PCR	1,789	66	3,664	32
Rapid antigen test	936	34	7,893	68
Month of swab				
July 2021	1,521	56	5,683	49
August 2021	1,204	44	5,874	51
Study site				
EN	10	0	39	0
ES	177	6	27	0
FR	96	4	69	1
HR	25	1	18	0
IE	20	1	169	1
NA	2,140	79	9,657	84
NL-CO	90	3	769	7
PT	14	1	69	1
RO	5	0	12	0
SC	148	5	728	6

COVID-19: coronavirus disease; ECDC: European Centre for Disease Prevention and Control; EN: England; ES: Spain; FR: France; HR: Croatia; IE: Ireland; NL-CO: the Netherlands community-based study; PT: Portugal; RO: Romania; SC: Scotland.

^a^ Among those commonly collected: diabetes, heart disease, chronic lung disease, immunodeficiencies, renal disease or liver disease.

Among controls, 7,893 of 11,557 (68%) and 936 of 2,725 (34%) were tested by rapid antigen test. In NL-CO 45 of 859 (5%), in ES 80 of 204 (39%), in FR 61 of 165 (37%), in NA 8,632 of 11,797 (73%) and in PT 11 of 83 (13%) were tested by rapid antigen test.

A total of 84% of controls had completed the primary COVID-19 vaccination schedule at the time of swabbing, compared with 59% of cases. Comirnaty was the most commonly used vaccine, with 66% of vaccinated controls and 60% of vaccinated cases receiving two doses.

Of the 2,190 cases presenting at the four study sites with sequencing results available, 131 (6%) were sequenced and 119 (91%) of these were infected with the SARS-CoV-2 Delta variant and its sublineages. All 20 viruses from HR, eight of nine viruses from IE, 88 of 99 viruses from NA and all three viruses from RO belonged to the Delta variant and its sublineages.

### COVID-19 vaccine effectiveness

The overall complete dose VE against symptomatic SARS-CoV-2 infection was 74% (95% CI: 70–77) among persons aged 30 years and older, adjusting for swab date, study site, age group, sex and chronic condition. The adjusted VE was 74% (95% CI: 69–79), 76% (95% CI: 71–80), 64% (95% CI: 48–75) and 63% (95% CI: 16–83) for those aged 30–44, 45–59, 60–74 and 75 years and older, respectively (Table 2). The VE for two doses of Comirnaty was 78% (95% CI: 75–81) and 67% (95% CI: 52–77) among 30–59-year-olds and those aged 60 and older, respectively. The VE for two doses of Vaxzevria was 66% (95% CI: 58–73) and 65% (95% CI: 48–76) among 30–59-year-olds and those aged 60 years and older, respectively. The VE for two doses of Spikevax was 91% (95% CI: 87–94) and 83% (85% CI: 64–92) among 30–59-year-olds and those aged 60 years and older, respectively. The VE of COVID-19 Vaccine Janssen was 52% (95% CI: 40–61) among 30–59-year-olds. VE was not computed for COVID-19 Vaccine Janssen among those aged 60 years and older, as all countries but one were excluded because of small numbers.

**Table 2:** Effectiveness of complete COVID-19 vaccination among participants in the primary care and community I-MOVE-COVID-19 and ECDC vaccine effectiveness study, by age group and vaccine product, Europe, July–August 2021 (n = 14,282)

Vaccine brand	Analysis type	Cases	Vaccinated cases	Controls	Vaccinated controls	Crude VE (95% CI)^a^	Adjusted VE (95% CI)^b^
Age group-specific analysis							
All vaccines^c^	≥ 30 years	2,725	1,606	11,557	9,706	74 (72–77)	74 (70–77)
	30–44 years	1,066	291	3,268	1938	75 (71–79)	74 (69–79)
	45–59 years	992	722	4,229	3,875	76 (71–80)	76 (71–80)
	60–74 years	410	346	2,412	2,277	64 (48–75)	63 (48–75)
	≥ 75 years	255	245	1,639	1,613	62 (16–83)	63 (16–83)
Comirnaty^d^	30–59 years	1,640	595	5,546	3,856	78 (75–80)	78 (75–81)
	≥ 60 years	449	375	2,735	2574	67 (54–76)	67 (52–77)
Vaxzevria^d^	30–59 years	1,141	151	2,294	639	66 (58–73)	66 (58–73)
	≥ 60 years	220	150	1,073	915	62 (45–74)	65 (48–76)
Spikevax^d^	30–59 years	1,071	38	2,268	596	91 (87–93)	91 (87–94)
	≥ 60 years	86	20	387	243	81 (66–90)	83 (64–92)
COVID-19 Vaccine Janssen^d^	30–59 years	1,136	217	2,199	621	46 (35–56)	52 (40–61)
	≥ 60 years	99	42	235	101	N/C^e^	
Chronic condition-specific analysis							
All vaccines^f^	Presence of chronic condition, 30–59 years	331	199	1,604	1,305	68 (58–76)	63 (50–73)
	Absence of chronic condition, 30–59 years	1,709	807	5,778	4,424	75 (72–78)	77 (73–80)
	Presence of chronic condition, ≥ 60 years	289	266	1,976	1,908	55 (24–74)	58 (27–76)
	Absence of chronic condition, ≥ 60 years	367	320	2,049	1,960	67 (51–78)	66 (49–78)
Comirnaty^g^	Presence of chronic condition, 30–59 years	257	125	1,190	891	72 (62–80)	69 (56–78)
	Absence of chronic condition, 30–59 years	1,383	470	4,329	2,946	79 (75–81)	80 (77–83)
Vaxzevria^g^	Presence of chronic condition, 30–59 years	143	20	381	89	54 (20–74)	50 (11–72)
	Absence of chronic condition, 30–59 years	997	131	1,904	543	67 (59–74)	68 (60–75)
Spikevax^g^	Presence of chronic condition, 30–59 years	136	5	464	167	94 (85–98)	94 (83–98)
	Absence of chronic condition, 30–59 years	935	33	1,802	429	89 (85–93)	90 (86–94)
COVID-19 Vaccine Janssen^g^	Presence of chronic condition, 30–59 years	168	46	423	132	25 (-15–50)	24 (-25–53)
	Absence of chronic condition, 30–59 years	968	171	1,774	489	50 (39–60)	57 (45–66)

CI: confidence interval; COVID-19: coronavirus disease; ECDC: European Centre for Disease Prevention and Control; EN: England; ES: Spain; FR: France; HR: Croatia; IE: Ireland; NL-CO: the Netherlands community-based study; PT: Portugal; RO: Romania; SC: Scotland; NA: Navarre; N/C: not calculated; VE: vaccine effectiveness.

^a^ Adjusted by study site and swab date.

^b^ Adjusted by study site, swab date, 10-year age group, presence of chronic condition, sex.

^c^ Because of small sample size, five controls were dropped from the 30–44 years (RO), four controls dropped from the 45–59 years (RO), and two cases dropped from the 75 years and older analysis (RO).

^d^ We included only countries where at least one study participant in the age group for analysis had received the vaccine under study. Comirnaty: EN, ES, FR, HR, IE, NA, NL-CO, PT, RO, SC; Vaxzevria: EN, ES, FR, HR (30–59-year-olds only), IE, NA, NL-CO, PT, SC; Spikevax: ES, FR, HR (30–59-year-olds only), IE (30–59-year-olds only), NA, NL-CO, PT, RO (60 years and older only), SC (30–59-year-olds only); COVID-19 Vaccine Janssen: ES, FR, HR, IE, NA, NL-CO, PT.

^e^ VE was not computed for COVID-19 Vaccine Janssen among those aged 60 and older, as only one country was left in the analysis.

^f^ Countries included in the analysis of 30–59-years-olds: ES, FR, HR, NA, NL-CO, PT, SC; countries included in the analysis of those 60 years and older: EN, ES, FR, IE, NA, NL-CO, RO, SC.

^g^ Because of small sample size, we dropped 22 controls from the Cominarty 'presence of chronic condition' 30–59 years analysis and five controls from the 'no chronic condition' analysis, nine controls and one case from the Vaxzevria 'presence of chronic condition' analysis and two controls each from the Spikevax and COVID-19 Vaccine Janssen 'presence of chronic condition' analysis.

The overall VE for those aged 30–59 years with presence of chronic condition was 63% (95% CI: 50–73) and without 77% (95% CI: 73–80) (p value for interaction term: 0.007). The VE for those aged 60 years and older with presence of chronic condition was 58% (95% CI: 27–76) and without 66% (95% CI: 49–78). Among those aged 30–59 years, the VE was 69% (95% CI: 56–78) and 80% (95% CI: 77–83), 50% (95% CI: 11–72) and 68% (95% CI: 60–75), 94% (95% CI: 83–98) and 90% (95% CI: 86–94), and 24% (95% CI: -25–53) and 57% (95% CI: 45–66), for those with and without presence of chronic condition for Comirnaty, Vaxzevria, Spikevax and COVID-19 Vaccine Janssen, respectively (Table 2). The sample size was too small to measure brand-specific VE stratified by presence of chronic condition among those aged 60 years and older.

**Figure 3:**
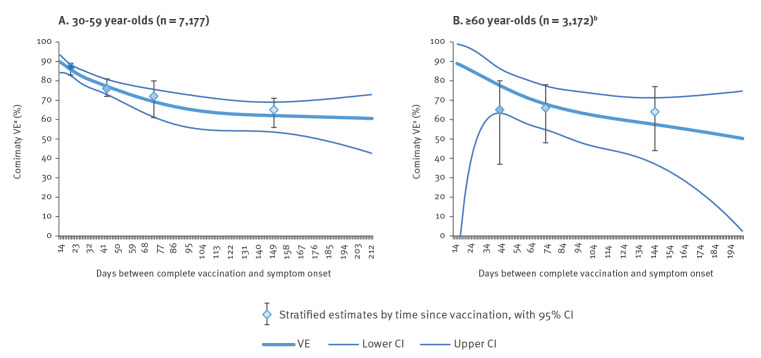
Effectiveness of Comirnaty vaccination among participants in the primary care/community I-MOVE-COVID-19 and ECDC vaccine effectiveness study by days between dose of complete vaccine and onset of symptoms and by age group, Europe, July–August 2021 (n = 10,370)

CI: confidence interval; ECDC: European Centre for Disease Prevention and Control; VE: vaccine effectiveness.

^a^ Adjusted by study site, age group, sex, date of swab and presence of chronic condition.

^b^ Sample size was too small to measure VE for 14–29 days for Comirnaty among those aged 60 years and older.

Comirnaty VE among those aged 30–59 by days between vaccination and onset of symptoms was 87% (95% CI: 83–89) at 14–29 days and 65% (95% CI: 56–71%) at ≥ 90 days (p value test for trend < 0.001) (Table 3). Using our modelling approach, the VE declined from 90% (95% CI: 84–94) at 14 days to 61% (95% CI: 43–73) at 213 days (Figure 3).

**Table 3:** Effectiveness of complete COVID-19 vaccination among participants in the primary care and community I-MOVE-COVID-19 and ECDC vaccine effectiveness study, by time since vaccination and vaccine product, Europe, July–August 2021 (n = 14,282 before exclusions)

Brand, age group and time since vaccination	Cases			Controls	Crude VE (95% CI)^a^	Adjusted VE (95% CI)^b^
Comirnaty, age 30–59 years^c^						
Unvaccinated	1,045			1,684	N/A	
Vaccinated 14–29 days	123			1,287	87 (84–89)	87 (83–89)
Vaccinated 30–59 days	261			1,584	75 (71–79)	76 (72–81)
Vaccinated 60–89 days	60			335	70 (59–78)	72 (61–80)
Vaccinated ≥ 90 days	151			647	66 (58–72)	65 (56–71)
Comirnaty, age ≥ 60 years^c^						
Unvaccinated	74		161		N/A	
Vaccinated 14–29 days	2		30		N/C	N/C
Vaccinated 30–59 days	32		425		67 (42–81)	65 (37–80)
Vaccinated 60–89 days	146		951		65 (49–76)	66 (48–78)
Vaccinated ≥ 90 days	192		1,159		66 (51–76)	64 (44–77)
Vaxzevria, age 30–59 years^d^						
Unvaccinated	990		1,655		N/A	
Vaccinated 14–29 days	21		107		71 (52–83)	72 (52–83)
Vaccinated 30–59 days	79		320		67 (56–75)	67 (57–75)
Vaccinated 60–89 days	42		162		64 (47–76)	65 (48–76)
Vaccinated ≥ 90 days	9		50		N/C	N/C
Spikevax, age 30–59 years^e^						
Unvaccinated	1,033		1,672		N/A	
Vaccinated 14–29 days	2		180		98 (92–100)	98 (93–100)
Vaccinated 30–59 days	19		285		91 (85–94)	91 (85–95)
Vaccinated 60–89 days	6		98		89 (75–96)	90 (76–96)
Vaccinated ≥ 90 days	11		33		N/C	N/C
COVID-19 Vaccine Janssen, age 30–59 years^f^						
Unvaccinated	919	1,578			N/A	
Vaccinated 14–29 days	19	61			N/C	N/C
Vaccinated 30–59 days	123	338			46 (32–57)	50 (36–62)
Vaccinated 60–89 days	70	205			45 (26–60)	52 (33–66)
Vaccinated ≥ 90 days	5	17			N/C	N/C

CI: confidence interval; EN: England; ES: Spain; FR: France; HR: Croatia; IE: Ireland; NA: Navarre; N/A: not applicable; N/C: not calculated (if stratum sample size was <100); NL-CO: the Netherlands community-based study; PT: Portugal; RO: Romania; SC: Scotland; VE: vaccine effectiveness.

^a^ Adjusted by study site and swab date.

^b^ Adjusted by study site, swab date, 10-year age group, presence of chronic condition and sex.

^c^ Comirnaty: EN (30–59-year-old analysis only), ES, FR, HR, IE, NA, NL-CO, PT, RO (60 years and older analysis only), SC. Because of small sample size, nine records were dropped from RO (30–59-year-old analysis) and 12 from EN (60 years and older analysis only).

^d^ Vaxzevria: EN, ES, FR, HR, IE, NA, NL-CO, PT, SC.

^e^ Spikevax: ES, FR, IE, NA, NL-CO, PT, SC.

^f^ COVID-19 Vaccine Janssen: ES, FR, HR, IE, NA, NL-CO, PT.

Comirnaty VE among those aged 60 years and older by days between vaccination and onset of symptoms was 65% (95% CI: 37–80) at 30–59 days and 64% (95% CI: 44–77%) at ≥ 90 days (Table 3). The sample size was too small to measure VE at 14–29 days (two vaccinated cases and 30 controls, respectively). Using the modelling approach the VE declined from 89% (95% CI: −22 to 99) at 16 days to 50% (95% CI: 2–75) at 203 days (Figure 3).

Vaxzevria VE among those aged 30–59 years by days between vaccination and onset of symptoms was 72% (95% CI: 52–83) at 14–29 days and 65% (95% CI: 48–76) at 60–89 days (Table 3). Spikevax VE among those aged 30–59 years by days between vaccination and onset of symptoms was 98% (95% CI: 93–100) at 14–29 days and 90% (95% CI: 76–96) at 60–89 days (Table 3). COVID-19 Vaccine Janssen VE among those aged 30–59 years by days between vaccination and onset of symptoms was 50% (95% CI: 36–62) at 30–59 days and 52% (95% CI: 33–66) at 60–89 days. The sample size was too small to measure COVID-19 Vaccine Janssen VE for 14–29 days and to measure VE among those vaccinated ≥ 90 days before symptom onset for all vaccines except Comirnaty, and modelling was not attempted. The sample size was also too small to measure VE by time since vaccination among those aged 60 years and older for these vaccine brands.

### Sensitivity analysis

Excluding the study site with self-selected participants (NL-CO), varying the imputed onset dates by 2 and 5 days, and restricting to those swabbed within 7 days of symptom onset, changed the VE point estimates by less than 3%. Excluding the study site providing the most cases and controls (NA) resulted in lower sample size and not all stratified VE could be estimated, particularly for older age groups. VE point estimates among those aged 30–59 years differed by ≤ 8% for all estimates. For those 60 years and older, we could only estimate VE for Comirnaty, which differed by 12%, although confidence intervals overlapped (67%; 95% CI: 52–77 and 79%; 95% CI: 54–91, including and excluding NA, respectively).

The VE point estimates in sensitivity analyses after excluding individuals with rapid tests were consistently lower (Supplementary Table S2 and S3). The absolute difference between overall VE estimates was 15% in the age group 30–44 years and 3% among those aged 45–59 years. After restricting to those tested by RT-PCR, we could no longer estimate VE among those aged 60 and older stratified into two age groups; the VE differed by 8% in that group. The VE estimates by age group (30–59 and ≥ 60 years) and vaccine brand differed by ≤ 10%, except for COVID-19 Vaccine Janssen among 30–59-year-olds, where the difference was 26%, but confidence intervals overlapped. The difference in VE estimates stratified by chronic condition differed by more than 10% among those with presence of chronic condition (confidence intervals overlapped) and among those without chronic condition for COVID-19 Vaccine Janssen (Supplementary Table S2 presents VE estimates by age group and by vaccine product restricted to those tested by RT-PCR only). The Comirnaty VE by time since vaccination had the same trends among individuals tested by RT-PCR only, although the final estimates were lower (see Supplementary Table S3 and Supplementary Figure S3 for VE estimates by time since vaccination restricted to those tested by RT-PCR only).

Varying the position of knots in time since vaccination analysis (equal intervals, different percentiles) changed the Akaike information criterion (AIC) by < 3 points among those aged 30–59 years and < 4 points among those 60 and older, and the functional forms remained the same.

## Discussion

In this multicentre test-negative design study, including 14,282 individuals, we measured VE of Comirnaty, Vaxzevria, Spikevax and COVID-19 Vaccine Janssen against symptomatic SARS-CoV-2 infection in July and August 2021, thus restricting the analysis to a period where Delta variant circulation dominated.

The VE was greater for mRNA vaccines than for the other vaccines among those aged 30–59 years, with Spikevax at 91%, Comirnaty at 78%, and Vaxzevria and COVID-19 Vaccine Janssen VE at 66% and 52%, respectively. Among those aged 60 years and older, VE was 67% (Comirnaty), 83% (Spikevax) and 65% (Vaxzevria). The sample size was too small to measure VE in this age group for COVID-19 Vaccine Janssen and confidence intervals overlapped for all other vaccine brands.

Owing to the observational nature of this study, comparison between vaccine brands should be made with caution, as different population groups (with varying levels of risk and exposure) may have been offered different vaccines at different times. To account for this, we attempted a further stratification of brand- and age-specific VE by time since vaccination, to better compare brand-specific VE, but greater sample size is needed for a full comparison. Nevertheless, our results suggest a reduction greater than 50% in symptomatic SARS-CoV-2 infection by all vaccine brands across all adults 30 years and older in July and August 2021 during dominant circulation of the Delta variant. However, those vaccinated earlier may belong to specific groups with different risks of exposure and susceptibility to the virus than those vaccinated late. Measuring age group and brand-specific VE by time since vaccination by vaccine cohort (vaccinated early, middle or late), although not possible in this study, would provide additional information.

The Comirnaty VE among those aged 60 years and older was lower than in our previous publication covering people aged 65 and older for the study period December 2020 to May 2021 (67% vs 87%, respectively) [16]. The differences in VE may be explained by the difference in predominant circulating variants between these periods (Alpha variant vs Delta variant) and potential waning of the effect of the vaccine over time.

Our estimates are also similar to the VE against symptomatic infection in other observational studies during circulation of the Delta variant. In the UK, while the VE point estimate of Comirnaty was higher (88% compared with our VE of 67–78%), the VE for Vaxzevria was similar (67% vs 65–66%) [22]. In Qatar, the VE of Comirnaty and Spikevax was lower (52% compared with our 67–78% and 73% compared with our 83–91%), although the study design differed from ours [23].

We observed a lower VE point estimate among those presenting at least one chronic condition compared with those without, both for 30–59-year-olds and those aged 60 years and older. Precision was low in the older age group, and the difference (14%) was only statistically significant among those aged 30–59 years. Brand-specific VE by chronic conditions among those aged 30–59 years suggest that for most brands, VE is higher among those without chronic conditions. Brand-specific VE by presence of chronic condition was not available for other age groups because of limited sample size. Small differences in VE against infection between those with and without underlying chronic conditions has been suggested in a Danish study [24], and specifically for immunocompromised people, but not for any chronic condition in a UK study [25], although VE was also reduced for immunocompromised people. Other studies did not observe differences in VE against infection among those with and without presence of chronic conditions [26,27]. Further studies presenting brand-specific VE by age group, including also estimates among those without chronic conditions as a comparator group and taking time since vaccination into account (as those with chronic conditions tend to be vaccinated earlier) are needed to add to the evidence of how the vaccines perform in different risk groups.

There was some evidence of decline in VE with time since vaccination among those aged 30–59 for Comirnaty (ranging from 87% at 14–29 days to 65% at ≥ 90 days). Modelling suggested the VE declined to 61% after 200 days. This is very similar to the results from a recent meta-analysis on duration of effectiveness of COVID-19 vaccines, showing a decline of 25 percentage points over 6 months [15]. Among those aged 60 years and older, VE point estimates differed minimally between 30 and 59 days and ≥ 90 days (63–66%), using an approach of measuring VE by time since vaccination by cut-offs, but precision was low. Modelling VE by time since vaccination as a continuous variable indicated some potential decline over time, but confidence intervals were also compatible with no decline. Sample size in this age group was too low to measure VE by time since vaccination for Spikevax and Vaxzevria. It is worth noting that this study did not include frail elderly people, e.g. those who present directly to hospital or residents in a care facility.

Using a modelling approach to measuring VE by days since vaccination provides added value compared with the stratified approach, as we do not lose information by transforming a continuous variable into a categorical one. However, the modelling approach may not be adequate in the tails of the model, because of sparse data, as can be seen by the very wide confidence intervals around the model among those aged 60 years and older. This may explain why the stratified estimates did not match the ones in the model up to 60 days between vaccination and symptom onset.

The decline of Comirnaty VE against infection for younger adults in the UK and of both mRNA vaccines for adults in Canada was smaller (both 12% compared with our 22% over a similar time since vaccination period) [14,28]. A larger decline in Comirnaty VE against infection with the Delta variant was observed among healthcare workers in the United States (US) (93% to 53% over 5 months) [29]. Similar to our study, no decline was observed for Vaxzevria among this age group in the UK and at most a 12% decline among adults in Canada. No decline was observed in the UK for Spikevax, however a 14% decline was observed in the US [30].

To disentangle the decline in VE by time since vaccination from variant-related reduction in VE, effects of vaccine brands given at different times and differences in age-specific VE, it is important to examine the data and measure VE by time since vaccination by age group and vaccine brand within a period of stable variant circulation. While this is possible to a certain extent in our study, sample size becomes limited and some results may be unreliable. We will endeavour to repeat these analyses going forward with larger sample size.

Study design-specific issues and the mechanism of protection of the vaccine can hide or exacerbate changes in VE by time since vaccination [31]. A false waning in the vaccine effect can be observed if the vaccine provides only partial protection, caused by differential depletion of susceptible people in unvaccinated and vaccinated groups [32]. In the context of a high baseline VE, which is what we observe here, the bias may be minimal [33].

In this study, five of 10 study sites used rapid antigen tests within their surveillance system and we included individuals in the analysis who had a rapid antigen test performed within 5 days of symptom onset. In a sensitivity analysis, VE estimates excluding those individuals with rapid antigen tests resulted in general in lower point estimates, although confidence intervals overlapped for all results. A particular difference in VE point estimates, however, was the overall VE among those aged 30–44 at 59% when excluding individuals with rapid antigen tests, compared with 74% when including them. These results suggest that there may be differences in testing behaviour in certain population groups that could be differential by case and vaccination status. Going forwards in the pandemic, recommendations for testing will change, overall and according to age and risk group, and it will be important to understand on an ongoing basis who is tested how and why. This is a challenge for researchers, particularly at primary care level.

Our study is subject to several limitations. While many study sites sequence the whole or partial genome of viruses, the proportion sequenced overall did not allow a variant-specific VE analysis. To overcome this limitation, we restricted the study period to July and August 2021, a period in which we know the Delta variant dominated in the European sites participating in our study. Among the 130 viruses sequenced, 91% belonged to the SARS-CoV-2 Delta variant, indicating that the vast majority of viruses in the study were likely to belong to the Delta variant. The proportion of viruses sequenced was small in our study, the main reason for this was the difficulty of being able to match the clinical-epidemiological data with the virological data.

The vaccine coverage was high in July and August 2021 in many European countries, particularly among older age groups. Apart from affecting our results in terms of precision, individuals who are unvaccinated may be different from vaccinated individuals in terms of exposure to the virus, thus violating a key precondition of VE studies [34]. Inclusion of confounders in the VE model can help adjust for different exposures. Including data on clinically extremely vulnerable people may help overcome confounding [35]. Confounding factors relating to behaviour are difficult to collect, although ongoing prospective cohort studies in special populations (e.g. healthcare workers) may help us understand what confounding to expect.

We used a multicentre study design and aimed to reduce heterogeneity with study sites adapting the same generic protocol. The study site NA dominated in terms of sample size, as it provided data from all symptomatic individuals tested using their comprehensive surveillance in electronic medical records/registries. Excluding NA from the analysis reduced precision, however, the point estimates for all analyses remained < 10% different in terms of absolute VE, with the exception of Comirnaty VE among people 60 years and older, where excluding NA resulted in a point estimate greater by 12%, but owing to sample size, confidence intervals overlapped.

Our study has several strengths. Selection bias can be a problem with observational studies, particularly in analyses involving electronic medical records. In our study, in all study sites but one, all or a systematic sample of individuals were selected for inclusion. The swabbing procedure was known, reducing the risk of selection bias. In addition, the test-negative design aims to minimise selection bias by adjusting for healthcare-seeking behaviour. Omitting the one study with self-selected individuals changed the VE by < 2% overall, by age group and vaccine brand.

## Conclusions

Our study provides evidence of 52–91% protection of a completed SARS-CoV-2 primary vaccination schedule against the Delta variant among those aged 30 years and older for four vaccine brands. For some vaccine brands, the results indicate a lower VE among those with a chronic condition among those aged 30–59. While our study suggests some waning of the vaccine effect with increased time since vaccination, we observed sustained protection of more than 60% up to 200 days after vaccination for Comirnaty. Further studies are needed to better analyse the observed decline in VE. We will continue the analysis of VE by days since vaccination and onset within the I-MOVE-COVID-19 and ECDC network study as more data come in. High complete vaccine coverage in the primary series of vaccination is important as it provides good direct protection of individuals against symptomatic SARS-CoV-2 infection as illustrated by this and other studies, as well as providing protection against severe disease.

## Supplementary Data

246000 Supplement
