# Supplementary material for: Effectiveness of complete primary vaccination against COVID-19 at primary care and community level during predominant Delta circulation in Europe: multicentre analysis, I-MOVE-COVID-19 and ECDC networks, July to August 2021
Source: Euro Surveill. 2022 May 26;27(21):2101104. doi: 10.2807/1560-7917.ES.2022.27.21.2101104 (PMC9137272; doi:10.2807/1560-7917.ES.2022.27.21.2101104)
Supplement: Supplement [file 21-01104_KISSLING_Supplement.pdf]

This supplementary material is hosted by *Eurosurveillance* as supporting information alongside the article “Effectiveness of complete primary vaccination against COVID-19 at primary care and community level during predominant Delta circulation in Europe: multicentre analysis, I-MOVE-COVID-19 and ECDC networks, July to August 2021” on behalf of the authors who remain responsible for the accuracy and appropriateness of the content. The same standards for ethics, copyright, attributions and permissions as for the article apply. Supplements are not edited by *Eurosurveillance* and the journal is not responsible for the maintenance of any links or email addresses provided therein.

Supplementary table 1: Characteristics of study sites in the I-MOVE-COVID-19 and ECDC primary care multicentre networks included in this analysis, July-August 2021 (n=10)

**Country**	**Study site**	**Comprehensive /sentinel / self-selected**	**Primary care physician (PC) / community (C)**	**Site of swab collection**	**Selection of patients** All (A), Systematic sample (S), Self-selected (SS)	**Vaccines used in study population**[a] Cominarty (C), Vaxzevria (V), Spikevax (S), Janssen (J)	**Source of all information** Electronic medical records (E), vaccine registry (V), GP interview (GP), patient questionnaire (P), other health databases, including national laboratory databases (H)	**Lab tests used** RT-PCR (PCR), rapid antigen test (RAT)
**Croatia**	HR	Sentinel	PC	At medical laboratory or by physician	A	C, V, S, J	E, V, GP	PCR
**England**	EN	Sentinel	PC	By physician	S	C, V	E, V	PCR
**France**	FR	Sentinel	PC	At medical laboratory or by physician	A	C, V, S, J	E, GP	PCR, RAT
**Ireland**	IE	Sentinel	PC	At COVID-19 testing centre	S	C, V, S	V, GP, H	PCR
**The Netherlands**	NL-CO	Self-selected	C	At COVID-19 testing centre	SS	C, V, S, J	P, E	PCR, RAT
**Portugal**	PT	Sentinel	PC	At COVID-19 testing centre	S	C, V, S	GP, H, V, E	PCR, RAT
**Romania**	RO	Sentinel	PC	By physician	S	C, V, S, J	E, GP	PCR
**Scotland**	SC	Sentinel	C	At COVID-19 testing centre	S	C, V	E, P	PCR
**Spain (3 regions)**	ES	Sentinel	PC	By physician	S (2 regions), A (1 region)	C, V	GP, E, V	PCR, RAT
**Spain (Navarra)**	NA	Comprehensive	PC	By physician	A	C, V, S, J	E, V	PCR, RAT

[a] Study population between 1 July and 31 August 2021. Other vaccines may have been used in these countries, but were not captured within this study

Supplementary Figure S1: Flowchart of exclusions for participants in the I-MOVE-COVID-19 and ECDC primary care multicentre network study, July-August 2021

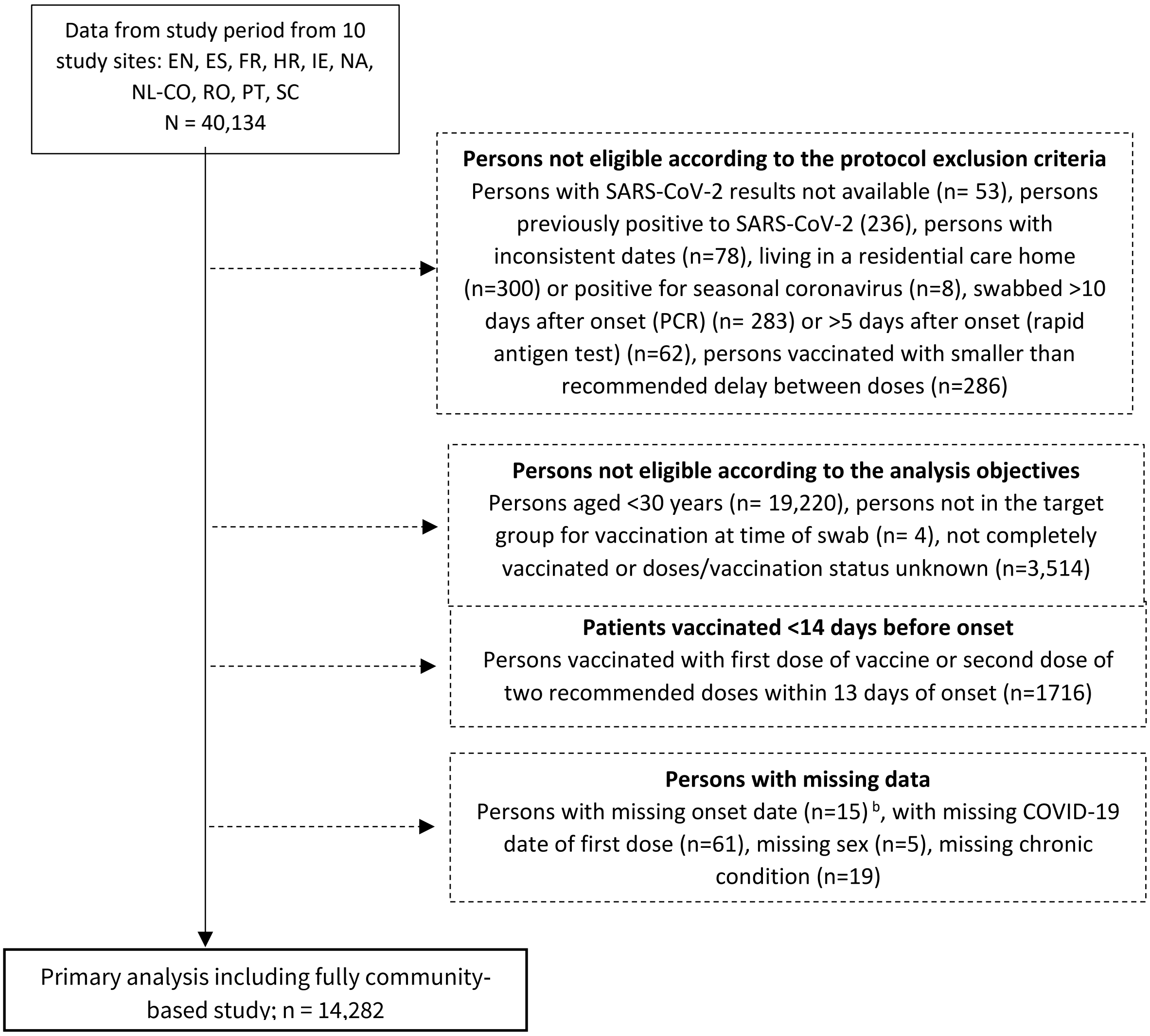

a In NA these patients were excluded prior to sending to the central hub and not included in the flowchart.

b Onset dates were imputed in NA, IE, PT.

Supplementary Figure S2: Completely vaccinated patients by case status and days between complete vaccination and symptom onset, I-MOVE-COVID-19 and ECDC primary care and community multicentre networks, July–August 2021

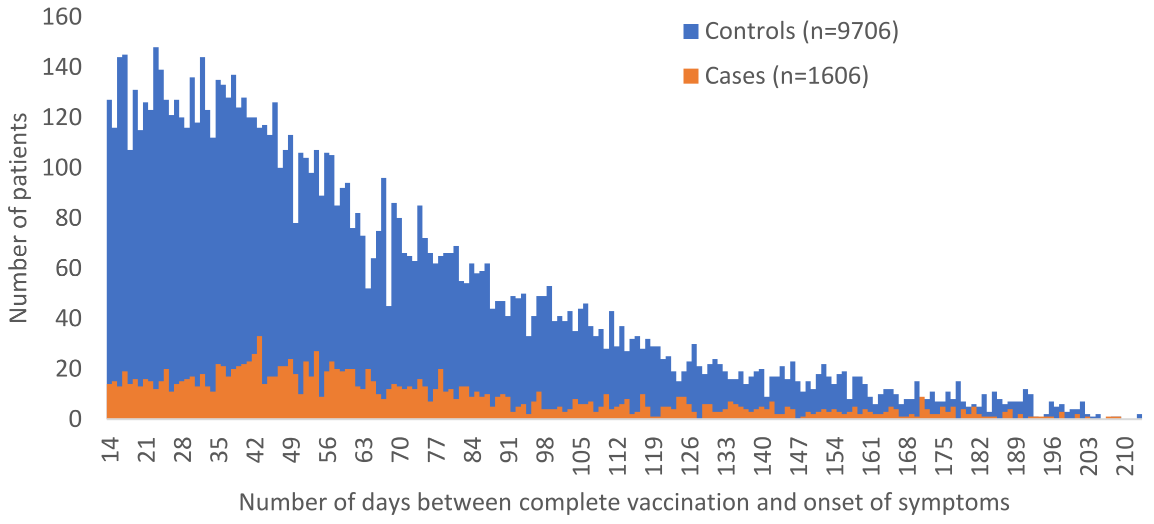

Table S2: Effectiveness of complete COVID-19 vaccination among participants in the primary care and community I-MOVE-COVID-19 and ECDC VE study, by age group and vaccine product, **restricted to those tested by RT-PCR only**, Europe, July–August 2021

Vaccine brand	Analysis type	Cases; vaccinated/ controls; vaccinated	Crude VE (95% CI)[a]	Adjusted VE (95% CI)[b]
**Age group-specific analysis**				
**All vaccines**[c]	Ages 30 years and older	1789;1276/3664;3222	66 (60-71)	65 (57-71)
	30–44 years	571;225/871;575	61 (49-69)	59 (47-69)
	45–59 years	701;572/1350;1259	73 (63-81)	73 (62-80)
	60+ years	517;479/1434;1385	58 (28–75)	55 (23–73)
**Comirnaty**[d]	30–59 years	936;461/1505;1112	69 (63-75)	70 (62-76)
	60 years and older	335;297/849;800	63 (36-79)	57 (24-76)
**Vaxzevria**[d]	30–59 years	534;121/677;327	57 (43-68)	59 (44-70)
	60 years and older	160;126/500;454	56 (22-75)	61 (30-78)
**Spikevax**[d]	30–59 years	495;32/552;177	84 (75-89)	85 (75-90)
	60 years and older	47;17/90;58	NC	NC
**COVID-19 Vaccine Janssen**[e]	30–59 years	537;172/475;179	9 (-22-32)	26 (-4-48)
	60 years and older[e]	60;34/46;20	NC	NC
**Chronic condition-specific analysis**				

**All vaccines**[f]	**Presence of chronic condition,** 30–59 years	204;148/416;363	53 (25-71)	38 (-4-63)
	**Absence of chronic condition**, 30–59 years	1050;642/1690;1387	65 (58-72)	68 (60-75)
	**Presence of chronic condition,** 60 years and older	220;209/591;576	43 (-39-76)	41 (-44-76)
	**Absence of chronic condition**, 60 years and older	286;263/818;787	63 (26-81)	61 (23-81)
**Comirnaty**	**Presence of chronic condition,** 30–59 years	149;93/304;251	62 (37-77)	51 (15-72)
	**Absence of chronic condition**, 30–59 years	787;368/1174;842	71 (63-76)	73 (66-79)
**Vaxzevria** [g]	**Absence of chronic condition**, 30–59 years	474;110/601;301	59 (45-70)	62 (47-73)
**Spikevax** [g]	**Absence of chronic condition**, 30–59 years	436;28/454;132	82 (71-89)	84 (73-90)
**COVID-19 Vaccine Janssen** [g]	**Absence of chronic condition**, 30–59 years	451;135/385;141	11 (-23-36)	33 (0-55)

CI: confidence interval; COVID-19: coronavirus disease; ECDC: European Centre for Disease Prevention and Control; EN: England; ES: Spain; FR: France; HR: Croatia; IE: Ireland; NL-CO: the Netherlands community-based study; PT: Portugal; RO: Romania; SC: Scotland; NA: Navarre; NC: not calculated; VE: vaccine effectiveness.

[a] Adjusted by study site, swab date.

[b] Adjusted by study site, swab date, 10-year age group, presence of chronic condition, sex.

[c] Six controls dropped from the 30–44 years (RO), four controls dropped from the 45–59 years (RO), and two cases dropped from the 75 years and older analysis (RO).

[d] Only countries included where at least one study participant in the age group for analysis had received the vaccine under study. Comirnaty: EN, ES, FR, HR, IE, NA, NL-CO, PT, RO, SC; Vaxzevria: EN, ES, FR, HR (30–59-year-olds only), IE, NA, NL-CO, PT, SC; Spikevax: ES, FR, HR (30–59-year-olds only), IE (30–59-year-olds only), NA, NL-CO, PT, RO (60 years and older only), SC (30–59-year-olds only); COVID-19 Vaccine Janssen: ES, FR, HR, IE, NA, NL-CO, PT

[e] VE was not computed for COVID-19 Vaccine Janssen among those aged 60 and older, as only one country was left in the analysis.

[f] Countries included in the 30–59 years old analysis: ES, FR, HR, NA, NL-CO, PT, SC; countries included in the 60 years and over analysis: EN, ES, FR, IE, NA, NL-CO, RO, SC

[g] Sample size too small to estimate VE among those with chronic condition.

Table S3: Effectiveness of complete COVID-19 vaccination among participants in the primary care and community I-MOVE-COVID-19 and ECDC VE study, by time since vaccination and vaccine product, **restricted to those tested by RT-PCR only**, Europe, July–August 2021

**Analysis by time since vaccination**			
**Brand, age group and time since vaccination**	**Cases / controls**	**Crude VE (95% CI)**[a]	**Adjusted VE (95% CI)**[b]
**Comirnaty, age 30–59 years**[c,d]			
Unvaccinated	475/387		
Vaccinated 14–29 days	87/348	80 (73-85)	82 (75-87)
Vaccinated 30–59 days	198/425	65 (54-73)	66 (55-75)

Vaccinated 60–89 days	46/98	59 (37-73)	63 (40-77)
Vaccinated ≥90 days	130/238	55 (41-66)	54 (38-66)
**Comirnaty, age 60+ years[c,d]**			
Unvaccinated	38/49		
Vaccinated 14–29 days	2/22	NC	NC
Vaccinated 30–59 days	21/147	68 (27-86)	68 (21-87)
Vaccinated 60–89 days	113/273	63 (31-80)	60 (20-80)
Vaccinated ≥90 days	158/349	56 (24-75)	46 (0-70)

CI: confidence interval; COVID-19: coronavirus disease; ECDC: European Centre for Disease Prevention and Control; EN: England; ES: Spain; FR: France; HR: Croatia; IE: Ireland; NL-CO: the Netherlands community-based study; PT: Portugal; RO: Romania; SC: Scotland; NA: Navarre; NC: not calculated; VE: vaccine effectiveness.

[a] Adjusted by study site, swab date.

[b] Adjusted by study site, swab date, 10-year age group, presence of chronic condition, sex.

[c] Comirnaty: EN (30–59-year-old analysis only), ES, FR, HR, IE, NA, NL-CO, PT, RO (60 years and older analysis only), SC

[d] VE by time since vaccination was not estimated for Vaxzevria, Spikevax and Janssen, as sample size was spares among several categories.

Supplementary Figure S3: Effectiveness of Comirnaty vaccination among participants in the primary care/community I-MOVE-COVID-19 and ECDC VE study by days between dose of complete vaccine and onset of symptoms, and by age group, **restricted to those tested by RT-PCR only**, Europe, July–August 2021

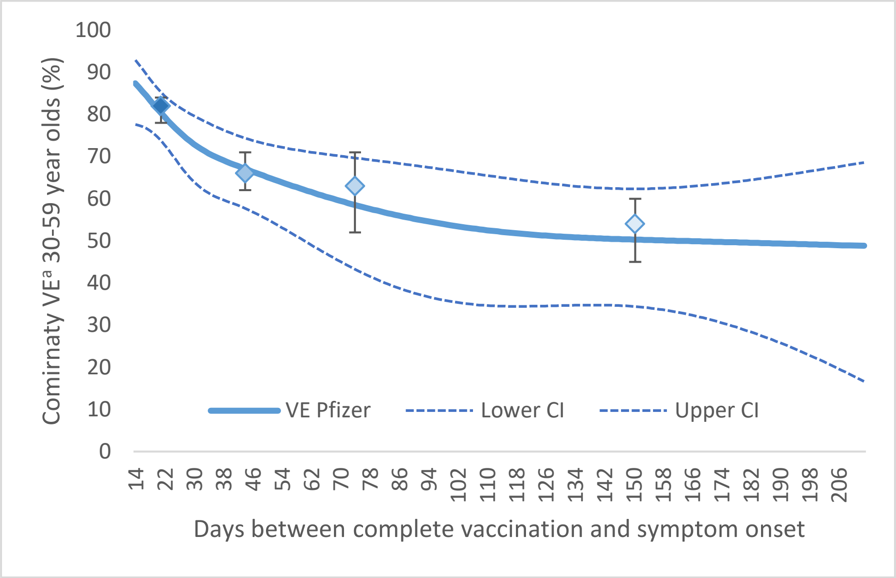

Comirnaty – 30–59-year olds

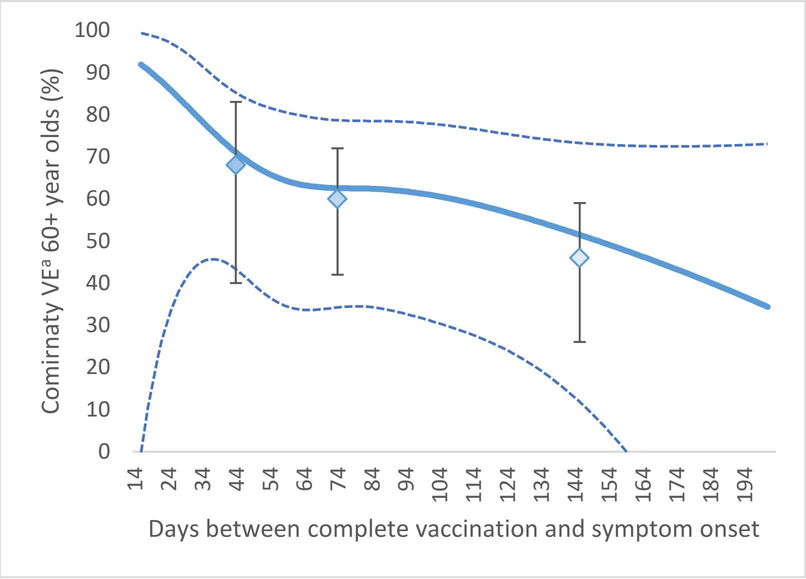

Comirnaty – 60 years and older

VE 14–29 days[b]
VE 30–59 days
VE 60–89 days
VE ≥90 days

CI: confidence interval; COVID-19: coronavirus disease; ECDC: European Centre for Disease Prevention and Control; VE: vaccine effectiveness.

[a]Adjusted by study site, age group, sex, date of swab and presence of chronic condition.

[b] Sample size was too small to measure VE for 14–29 days for Comirnaty among those aged 60 years and older
